# HipA-Mediated Phosphorylation of SeqA Does not Affect Replication Initiation in *Escherichia coli*

**DOI:** 10.3389/fmicb.2018.02637

**Published:** 2018-11-02

**Authors:** Leise Riber, Birgit M. Koch, Line Riis Kruse, Elsa Germain, Anders Løbner-Olesen

**Affiliations:** ^1^Section for Functional Genomics, Department of Biology, Center for Bacterial Stress Response and Persistence, University of Copenhagen, Copenhagen, Denmark; ^2^Laboratoire de Chimie Bactérienne, Université Aix-Marseille, CNRS, Marseille, France

**Keywords:** *E. coli*, SeqA protein, phosphorylation, HipA kinase, initiation synchrony, minimal inter-initiation time

## Abstract

The SeqA protein of *Escherichia coli* is required to prevent immediate re-initiation of chromosome replication from *oriC*. The SeqA protein is phosphorylated at the serine-36 (Ser36) residue by the HipA kinase. The role of phosphorylation was addressed by mutating the Ser36 residue to alanine, which cannot be phosphorylated and to aspartic acid, which mimics a phosphorylated serine residue. Both mutant strains were similar to wild-type with respect to origin concentration and initiation synchrony. The minimal time between successive initiations was also unchanged. We therefore suggest that SeqA phosphorylation at the Ser36 residue is silent, at least with respect to SeqA's role in replication initiation.

## Introduction

In *Escherichia coli* the DnaA initiator protein binds ATP and ADP with equal affinity (Sekimizu et al., 1987). DnaA binds three high-affinity sites in the origin, *oriC*, throughout the cell cycle irrespective of the bound nucleotide. The relative amounts of DnaA^ATP^ and DnaA^ADP^, respectively fluctuate during the cell cycle with the DnaA^ATP^/DnaA^ADP^ ratio peaking at initiation (Kurokawa et al., 1999). This results in binding of a number of additional DnaA binding sites of low affinity and with a preference for DnaA^ATP^ (Skarstad and Katayama, 2013; Leonard and Grimwade, 2015; Katayama et al., 2017). This induces origin opening, allows for helicase loading and replisome assembly.

Immediate re-initiation of new and hemimethylated origins is prevented by SeqA-binding to 11 GATC sites located within the minimal *oriC* (Campbell and Kleckner, 1990; Lu et al., 1994; Boye et al., 2000). The binding of SeqA to the origin prolongs the duration of the DNA hemi-methylated phase; a process called sequestration. Sequestration lasts approximately one-third of a cell cycle where re-initiation is prevented by SeqA denying DnaA^ATP^ access to GATC-containing low affinity DnaA boxes in *oriC* (Nievera et al., 2006). The sequestration period allows the cells to distinguish between “old” and “new” origins, and provides a time window where the DnaA^ATP^ level is lowered by RIDA (Kato and Katayama, 2001) and DDAH (Kasho and Katayama, 2013). Sequestration is finally terminated when GATC sequences within *oriC* become fully methylated by Dam methyltransferase.

In *seqA* mutant cells the sequestration period is shortened or absent (von Freiesleben et al., 2000), re-initiations occur frequently leading to over-initiation, and replication initiation becomes highly asynchronous (Lu et al., 1994). Conversely, excess SeqA protein prolongs the sequestration period, delays initiation, but does not affect initiation synchrony (Fossum et al., 2003; Charbon et al., 2011).

The SeqA protein contains two functional domains, an N-terminal oligomerization domain (SeqA-N; residues 1–33) and a C-terminal DNA-binding domain (SeqA-C; residues 65–181), which are joined by a flexible linker (residues 34–64; Chung et al., 2009). The interaction of SeqA with DNA occurs mainly in the major groove of the hemimethylated GATC sequences (Guarné et al., 2002), and data have suggested that two adjacent GATC sequences, up to 31 bp apart, interacting with the SeqA dimer are sufficient for strong binding (Guarné et al., 2005).

Recently, a stable isotope labeling by amino acids in cell culture (SILAC)-based quantitative phosphoproteomic approach combined with high-resolution mass spectrometry identified residue serine-36 (Ser36) in SeqA as a direct phosphorylation target for the kinase activity of the high persister protein A, HipA (Semanjski et al., 2018). HipA is an eukaryotic-like serine-threonine protein kinase that induces the stringent response, inhibits cell growth and confers cellular persistence through phosphorylation and inactivation of the glutamyl-tRNA-synthetase, GltX (Germain et al., 2013; Kaspy et al., 2013; Semanjski et al., 2018). The *hipA* gene constitutes a type II TA module with the adjacent upstream *hipB* gene, encoding the HipB antitoxin. HipB interacts directly with HipA to form a protein complex that represses the *hipBA* operon through binding to operators in the *hipBA* promoter region (Black et al., 1994), thereby counteracting the negative effect on cell growth caused by even low amounts of wild-type HipA (Korch and Hill, 2006).

It is not known whether phosphorylation at residue Ser36 of SeqA affects the activity and function of SeqA. Adding a phosphate group with negative charge to a protein, can promote changes in the structural conformation by altering the interactions with nearby amino acids. This might activate or inhibit the activity of the protein (Chao et al., 2014) or result in function modifications (Johnson and Barford, 1993).

Here, we tested the effect of Ser36 phosphorylation of SeqA on chromosome replication initiation. Two variants of SeqA were constructed, in which the Ser36 residue was either mutated to alanine (S36A) or aspartic acid (S36D). The S36A mutation impairs Ser36 phosphorylation, whereas the S36D mutation mimics the conformation of Ser36 phosphorylated SeqA (i.e., phospho-mimetic; (Arany et al., 2013). As both *seqA* mutants were similar to the wild-type with respect to synchrony and length of the sequestration period, our data suggest that HipA-mediated Ser36 phosphorylation of SeqA constitutes a neutral effect on the role of SeqA in *E. coli* replication initiation.

## Materials and methods

### Media and growth conditions

Cells were grown in AB minimal medium (Clark and Maaløe, 1967) supplemented with 1 μg/ml thiamine, 0.2% glucose and 0.5% casamino acids (glucose-CAA medium). When necessary, antibiotic selection was maintained at the following final concentrations: kanamycin, 50 μg/ml; chloramphenicol, 20 μg/ml; tetracycline, 10 μg/ml; ampicillin, 150 μg/ml. All cells were cultured at 37°C, except when otherwise indicated. Cell growth was monitored by measuring optical density at 450 nm (OD_450_).

### Bacterial strains

All strains used were derived from *E. coli* K-12 MG1655 (F^−^, λ^−^, *rph-1*; Guyer et al., 1981) and are listed in Table 1. The Δ*hipBA::frt::kan::frt* (Germain et al., 2013) and *dnaA46 tnaA600::*Tn*10* (Kogoma and von Meyenburg, 1983) alleles were moved by P1-phage-mediated transduction (Miller, 1972). To construct the chromosomal *seqA* mutant strains (*seqA*_*S*36*A*_ and *seqA*_*S*36*D*_, respectively), base substitutions were made in the codon for Ser36 (5′-TCC-3′ to 5′-**G**CC-3′; Ser(S) to Ala(A), and 5′-TCC-3′ to 5′-**GA**C-3′; Ser(S) to Asp(D), respectively) using splicing by overlap extension (SOEing) polymerase chain reaction (PCR) (Horton et al., 1989). All primers are listed in Table 2. For each *seqA* variant two initial PCR products of the MG1655 chromosome were generated. 1) The lower region of the *seqA* gene, spanning residues 27–181, was amplified using primers “SeqA_down_bw_XmaI” and either “SeqA_pos36_SA_fw” or “SeqA_pos36_SD_fw”. 2) The upper region of the *seqA* gene was amplified using primers “SeqA_up_fw_SacI” and “SeqA_intern_bw” that generates a fragment with an overlap of 21 bp with the *seqA* downstream PCR product. A secondary amplification was performed using equimolar ratios of the two PCR products as template, and the oligonucleotides, SeqA_down_bw_XmaI, and SeqA_up_fw_SacI, as primers. The resulting PCR fragments were digested with XmaI and SacI, and cloned into the same sites of the 3.9 kb suicide vector, pRUC1437, a derivative of pSW29T (Demarre et al., 2005), carrying the *aph* gene encoding kanaymicn resistance, and the *sacB* gene. The resulting plasmids were transformed into strain S17-1 (*recA thi pro hsdR*^−^*M*^+^ RP4-2 Tc::Mu-Km::Tn7 λ*-pir* lysogen Tp^R^ Sm^R^; (Simon et al., 1983) before being transferred into ALO 2956 cells by conjugation. Selection of exconjugants carrying the chromosomally integrated recombinant suicide plasmids as well as subsequent sucrose-mediated selection for loss of the *sacB* gene (i.e., loss of suicide vector sequences; (Donnenberg and Kaper, 1991), leaving either a wild-type or a mutant variant of the *seqA* gene on the MG1655 *lacIZYA::cat* chromosome, was performed as described previously (Riber et al., 2009). Chromosomal *seqA* mutant strains were verified by DNA sequencing of PCR fragments amplified from the *seqA* region using DNA oligonucleotides, “SeqA_chr_fw” and “SeqA_chr_bw,” as primers.

**Table 1:** Bacterial strains.

**Strains**	**Relevant genotype**	**Plasmid**	**Reference/Source**
MG1655	F^−^, λ^−^, *rph-1*	None	(Guyer et al., 1981)
ALO2956	*lacIZYA::cat^*a*^*	None	This work
ALO3758	Δ*seqA*^b^	None	(Riber et al., 2009)
ALO5105	Δ*hipBA::frt::kan::frt*^b^	None	(Germain et al., 2013)/This work
ALO5695	*seqA_*S*36*D*_*^b^	None	This work
ALO5945	*seqA_*S*36*A*_*^b^	None	This work
ALO6136	*dnaA46 tnaA600::*Tn*10* ^b^	None	This work
ALO6138	*dnaA46 tnaA600::*Tn*10* Δ*seqA*^b^	None	This work
ALO6141	*dnaA46 tnaA600::*Tn*10 seqA_*S*36*A*_*^b^	None	This work
ALO6143	*dnaA46 tnaA600::*Tn*10 seqA_*S*36*D*_*^b^	None	This work
ALO5090	Δ*seqA*^b^	pFH2102	This work
ALO5093	Δ*seqA*^b^	pMAK7	This work
ALO5095	Δ*seqA*^b^	pLR75	This work
ALO5101	Δ*seqA*^b^	pLR77	This work

a *Genotype otherwise as MG1655*.

b *Genotype otherwise as ALO2956*.

**Table 2:** Primers.

**Name**	**Sequence**
SeqA_down_bw_XmaI	5′-GGCGGCCCCGGGTTTGTCCTTTGTCTGCAACG
SeqA_up_fw_SacI	5′-GGCGGCGAGCTCCAGCTAAGACACTGCACTGG
SeqA_intern_bw	5′-CAACATACGCCGTAAAATGTC
SeqA_pos36_SA_fw	5′-GACATTTTACGGCGTATGTTGAAATTTGCCGCCGCA TCACAGCCTGCTGCTCCG
SeqA_pos36_SD_fw	5′-GACATTTTACGGCGTATGTTGAAATTTGACGCCGCA TCACAGCCTGCTGCTCCG
SeqA_chr_fw	5′-CCATTGTGCCACAGGGCTGCAAC
SeqA_chr_bw	5′-GCACTGCCACGGTGACCGGAAG
SeqA_up_fw_EcoRI	5′-GGCGGCGAATTCCAGCTAAGACACTGCACTGG
SeqA_down_bw_HindIII	5′-GGCGGCAAGCTTTTTGTCCTTTGTCTGCAACG

### Plasmids

All plasmids used are listed in Table 3. Plasmids pLR77 and pLR75 were constructed by PCR amplifying the *seqA* variant genes (including the native *seqA* ribosome binding site) from MG1655 *lacIZYA::cat* cells carrying either the *seqA*_*S*36*A*_ or *seqA*_*S*36*D*_ chromosomal genes (see above), respectively, using DNA oligonucleotides, SeqA_up_fw_EcoRI and SeqA_down_bw_HindIII, as primers. The resultant PCR fragments were digested with EcoRI and HindIII and inserted downstream the IPTG inducible *lacP*_*A*1−04/03_ promoter (Lanzer and Bujard, 1988) of plasmid pFH2102 (von Freiesleben et al., 2000), cut with the same enzymes. The inserted *seqA* mutant genes were later verified by DNA sequencing.

**Table 3:** Plasmids.

**Plasmid**	**Relevant genotype**	**Reference/Source**
pBR322	*bla, tet*	(Bolivar et al., 1977)
pFH2102	*ori*-pBR322, *lacP_*A*1/04−03_, lacI, bla*	(von Freiesleben et al., 2000)
pMAK7	*ori*-pBR322, *lacP_*A*1/04−03_-seqA, lacI, bla*	(von Freiesleben et al., 2000)
pLR75	*ori*-pBR322, *lacP_*A*1/04−03_-seqA_*S*36*D*_, lacI, bla*	This work
pLR77	*ori*-pBR322, *lacP_*A*1/04−03_-seqA_*S*36*A*_, lacI, bla*	This work

### Flow cytometry and cell cycle analysis

Exponentially growing cells (OD_450_ = 0.15–0.30) were treated with rifampicin (300 μg/ml; SERVA Electrophoresis GmbH) and cephalexin (36 μg/ml; Sigma-Aldrich) to inhibit initiation of DNA replication and cell division, respectively (Løbner-Olesen et al., 1989). Incubation continued for a minimum of 4 h at 37°C to allow completion of ongoing rounds of replication. Cells were fixed in 70% ethanol and stained with 90 μg/ml mithramycin (SERVA Electrophoresis GmbH) and 20 μg/ml ethidium bromide (Sigma-Aldrich) as described (Løbner-Olesen et al., 1989). Flow cytometry was performed as previously described (Løbner-Olesen et al., 1989) using an Apogee A10 instrument (Apogee, Inc.). For all samples a minimum of 50.000 cells were analyzed. Numbers of origins per cell and relative cell mass were determined as previously described (Løbner-Olesen et al., 1989).

### Immunoblot procedure

Samples of 2 ml of exponentially growing cells (OD_450_ = 0.3–0.4) were harvested. Proteins were separated by SDS-PAGE and SeqA protein detected by Western blot using rabbit antiserum raised against SeqA protein (Torheim et al., 2000) as previously described (Riber and Lobner-Olesen, 2005). The membrane was scanned using a 230 V GenoView imaging system equipped with a UV transilluminator (VWR). Quantification was done using the ImageJ software.

### Multiple sequence alignment analysis

Multiple alignment analysis of SeqA amino acids sequences was performed in the MEGA version 7.0.26 software (Kumar et al., 2016) using the default settings of the integrated ClustalW algorithm (Larkin et al., 2007). Selected species including SeqA protein accession numbers were: *Escherichia coli* K-12 (accession: AAA19855.1), *Vibrio cholerae* (accession: AOY47782.1)*, Pasteurella multocida* PM70 (accession: AAK02440.1), *Haemophilus influenzae* Rd (accession: NP_438362.1), *Yersinia enterocolitica* (accession: CNB62546.1), *Serratia marcescens* (accession: KFL03527.1), *Actinobacillus pleuropneumoniae* (accession: SQF64393.1), and *Glaesserella parasuis* (accession: STO80764.1).

## Results

### Changing the SeqA Ser36 residue mainly affects the linker region of SeqA

In order to determine any putative role of SeqA phosphorylation, we generated two mutations at the chromosomal codon 36 of *seqA*. In one strain, the codon for Ser36 was replaced with that of an aspartic acid (*seqA*_*S*36*D*_). The SeqA_S36D_ mimics the conformation of Ser36 phosphorylated SeqA (Arany et al., 2013). In a second strain the codon for Ser36 was replaced with that of an alanine (*seqA*_*S*36*A*_). The resulting protein, SeqA_S36A_, is phosphorylation impaired at position 36 (Arany et al., 2013).

**Figure 1:**
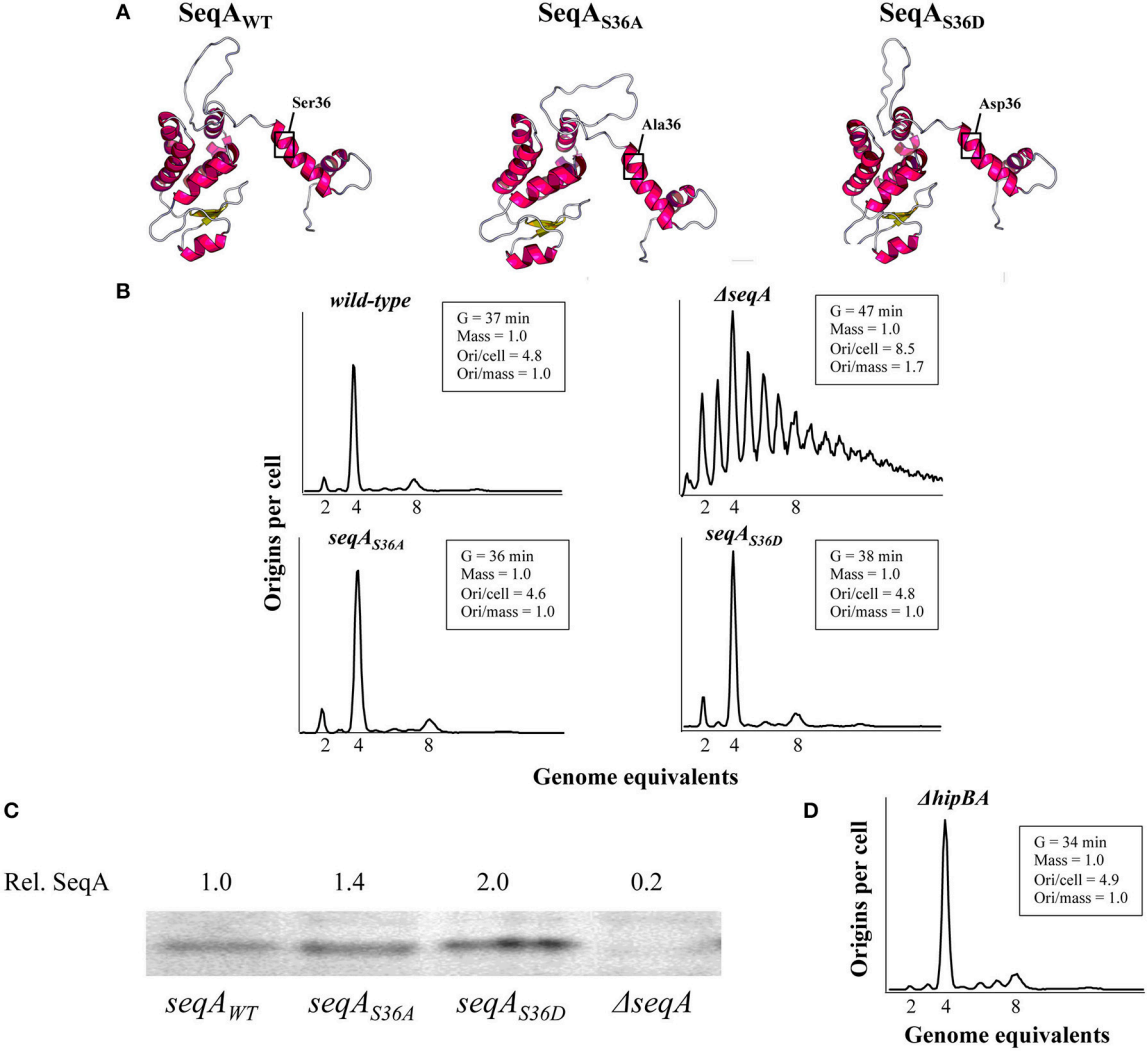
Replication initiation is not affected by the *seqA*_*S*36*A*_ and *seqA*_*S*36*D*_ mutations. **(A)** Prediction of tertiary structures of SeqA, SeqA_S36A_ and SeqA_S36D_ proteins using the RaptorX Structure Prediction web server (Källberg et al., 2012). **(B)** Wild-type, *seqA*_*S*36*A*_*, seqA*_*S*36*D*_, and Δ*seqA* cells were grown at 37°C in AB minimal medium supplemented with glucose and casamino acids. Cells were treated with rifampicin and cephalexin prior to flow cytometric analysis. Cell cycle parameters are shown in the insert. “Ori/cell” represents the average number of origins per cell, whereas “Ori/mass” represents the origin concentration. “Mass” and “Ori/mass” measures are relative to wild-type cells. **(C)** SeqA protein content determined by Western blot analysis. All quantifications are relative to wild-type cells. The relevant *seqA* genotype is indicated on the figure. **(D)** HipBA deficient cells were grown and subjected to flow cytometric analysis as described in **(B)** above.

We used the RaptorX web server (Källberg et al., 2012) to predict the tertiary structures (Peng and Xu, 2011; Ma et al., 2012) of the wild-type SeqA and the SeqA_S36A_ and SeqA_S36D_ proteins. This revealed a significant level of resemblance (Figure 1A). By pairwise and multiple structural alignments of the SeqA protein variants, TMScore values above 0.9 were obtained, illustrating a significantly increased likelihood (>90% of chance) that the proteins pairwise and all together share similar folds, RaptorX Structure Alignment Server; (Wang et al., 2011, 2013), with SeqA and SeqA_S36D_ being structurally most alike [TMScore (WT vs. S36D) = 0.96]. The structural differences caused by changing the Ser36 residue seem to affect only the flexible linker region between SeqA-N and SeqA-C (Figure 1A; Chung et al., 2009).

### Replication initiation is not affected by *seqA*_*s*36*A*_ and *seqA_*s*36*D*_* mutations

We used flow-cytometry to determine cell cycle parameters of wild-type and *seqA* mutants. The two *seqA* mutants grew with similar doubling times as wild-type cells in minimal medium supplemented with glucose and casamino acids, whereas cells deficient in SeqA grew with an ~30% increased doubling time relative to that of wild-type cells (Figure 1B). Following treatment with rifampicin and cephalexin, wild-type, *seqA*_*S*36*A*_ and *seqA*_*S*36*D*_ cells were similar and contained mainly 2, 4, or 8 fully replicated chromosomes, indicative of initiation synchrony (Skarstad et al., 1986). As the average cell mass and numbers of origins per cell were similar, so was the origin concentration between these three cell types. SeqA deficient cells showed an asynchronous initiation phenotype with an increased average number of origins, which illustrates a lost ability to negatively regulate replication initiation. The average cell mass was similar to that of wild-type cells resulting in an increased origin concentration (Figure 1B).

Because the SeqA_S36A_ and SeqA_S36D_ protein levels were comparable or slightly elevated relative to that of wild-type SeqA protein (Figure 1C), these data altogether suggest that phosphorylation of SeqA at position 36 has little influence on its activity in replication initiation control. This was further corroborated by analyzing cells deficient in the HipA kinase, i.e., with a knock-out of the *hipA* gene. Here we found that Δ*hipBA::kan* mutant cells displayed similar cell cycle parameters as wild-type cells (Figure 1D).

### Overproduction of SeqA, SeqA_S36A_, or SeqA_S36D_ proteins all restore initiation synchrony in Δ*seqA* mutant cells

We proceeded to examine whether overexpression of wild-type and mutant SeqA proteins could reveal any difference in activity among the phospho-impaired (S36A), phospho-mimetic (S36D) and wild-type SeqA proteins.

**Figure 2:**
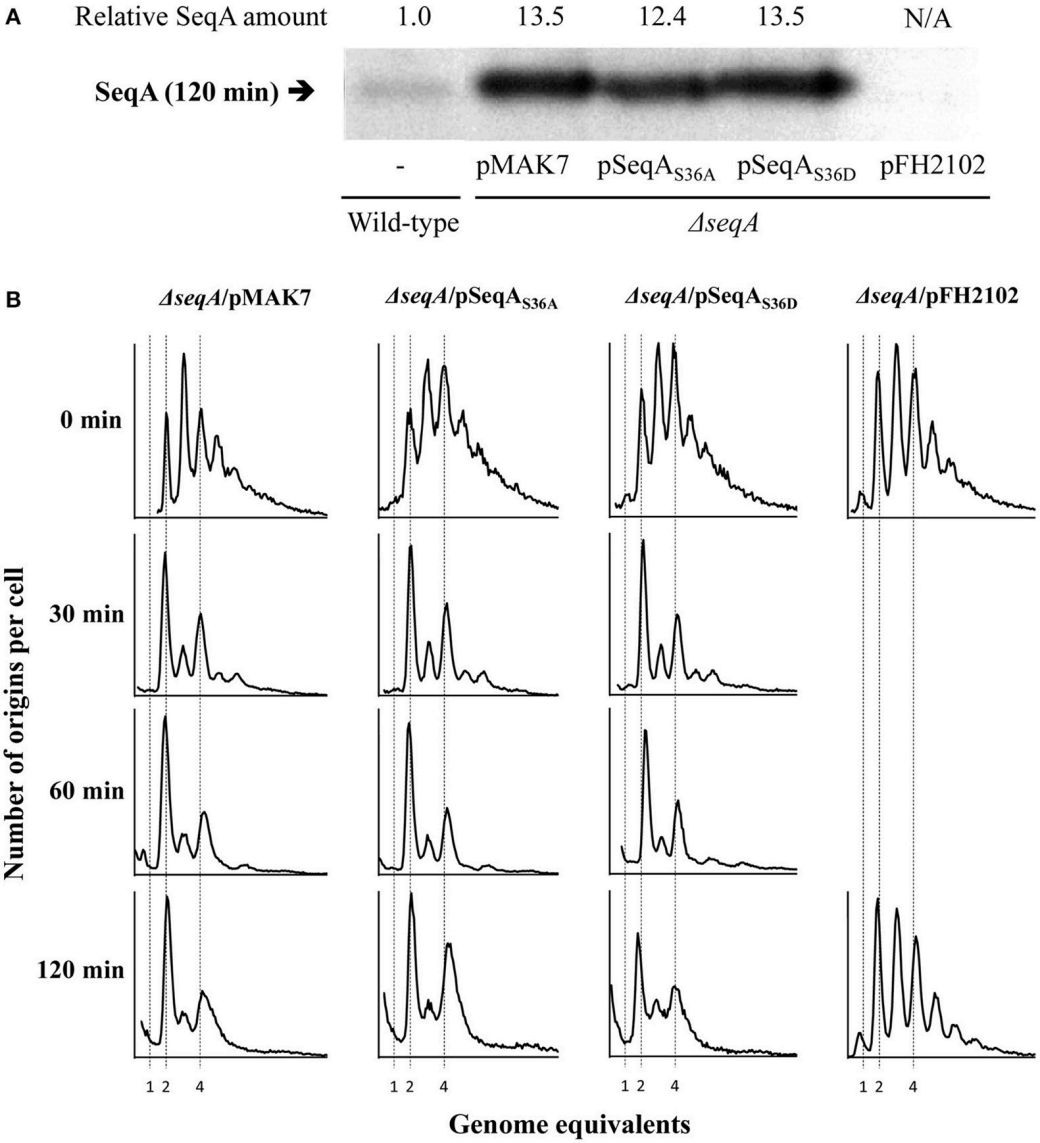
Similar effects of SeqA_WT_, SeqA_S36A_, or SeqA_S36D_ protein overproduction on replication initiation. SeqA deficient (Δ*seqA*) cells carrying the SeqA expression plasmids, pMAK7 (pLac-*seqA*), pLR77 (pLac-*seqA*_*S*36*A*_), pLR75 (pLac-*seqA*_*S*36*D*_), and pFH2102 (vector) were grown exponentially at 37°C in AB minimal medium supplemented with glucose and casamino acids. At time, T = 0 min (top panel), IPTG was added to a final concentration of 1 mM, and samples were subsequently removed at the indicated time points. **(A)** SeqA immunoblot sampled at 120 min. A sample of wild-type cells (without plasmid) is included to allow for relative quantification of SeqA levels. **(B)** Samples were taken at 0, 30, 60, and 120 min following IPTG induction and treated with rifampicin and cephalexin prior to flow cytometric analysis.

We expressed the *seqA, seqA*_*S*36*A*_, and *seqA*_*S*36*D*_ genes from the IPTG-inducible *lacP*_*A*1/04−03_ promoter in SeqA deficient cells. Exponentially growing cells were induced with 1 mM IPTG at time 0 min (T = 0 min). Immunoblot analysis of cells sampled at 120 min following the addition of IPTG indicated that all SeqA proteins were expressed to comparable levels corresponding to an ~12- to 14-fold increase in SeqA level relative to wild-type cells (Figure 2A). Both wild-type and mutant SeqA proteins complemented Δ*seqA* cells to the same extent when produced from a plasmid. Cells containing mainly two or four origins, indicative of initiation synchrony, dominated the population already after 30 min induction of the *seqA* variant genes (Figure 2B). A larger increase in mutant SeqA proteins (T = 120 min) resulted in no significant asynchrony relative to wild-type (Figure 2B). This is in agreement with earlier data on SeqA overproduction (Fossum et al., 2003).

### The minimal time between successive initiations is not altered by *seqA*_*S*36*A*_ and *seqA_*S*36*D*_* mutations

Changes in the duration of sequestration by increasing or decreasing the level of Dam methylase (von Freiesleben et al., 2000) or by increasing the SeqA level (Charbon et al., 2011) were previously found to have relatively modest effects on the cell cycle relative to complete loss of sequestration. We therefore proceeded to determine whether the SeqA mutant proteins affected the length of the sequestration period, defined as the minimal time between successive initiations (von Freiesleben et al., 2000).

**Figure 3:**
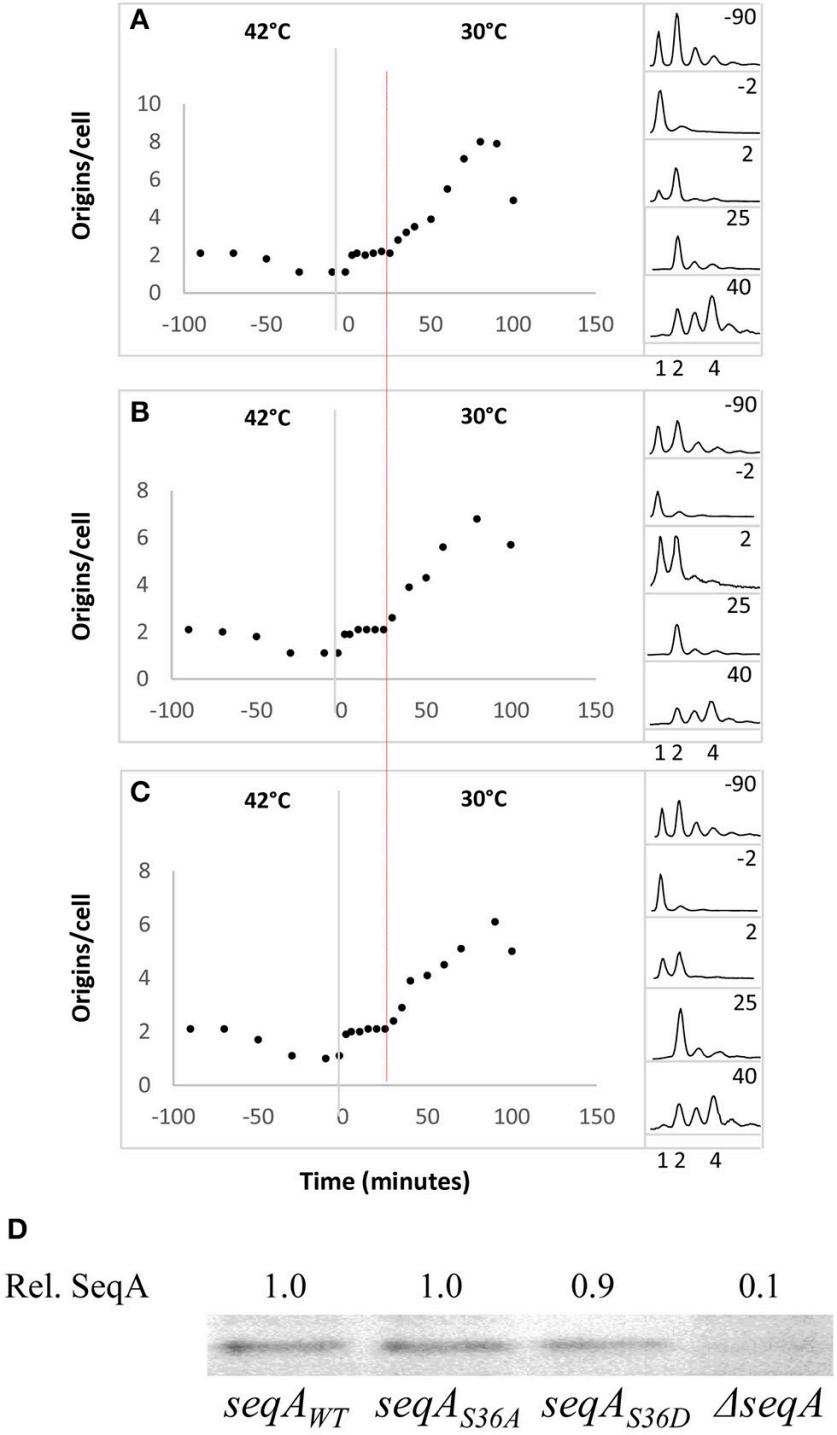
The *seqA*_*S*36*A*_ and *seqA*_*S*36*D*_ mutations do not change the minimal time between successive initiations. *dnaA46* **(A)**, *dnaA46 seqA*_*S*36*A*_ **(B)**, and *dnaA46 seqA*_*S*36*D*_ **(C)** cells were grown exponentially at 30°C in AB minimal medium supplemented with glucose and casamino acids. At time *T* = −90 min the cultures were shifted to the non-permissive temperature (42°C) and at time *T* = 0 min (illustrated by the gray vertical lines) shifted back to 30°C. At the times indicated samples were removed for treatment with rifampicin and cephalexin prior to flow cytometric analysis. The median (the value above and below which 50% of the distribution can be found) was used as a robust measure of the central tendency of individual cells (von Freiesleben et al., 2000) and is plotted as origins per cell. Replication resumes by 2 min (1 to 2 ori/cell). The red vertical indicates a second roundof firing giving rise to 4 ori/cell **(A–C)**. The panels on the right-hand side of the figure show selected DNA histograms for rifampicin-cephalexin treated cultures **(D)**. SeqA protein content determined by Western blot analysis for wild-type, *seqA*_*S*36*A*_, *seqA*_*S*36*D*_, or Δ*seqA* cells carrying the *dnaA46* allele. All quantifications are relative to SeqA^+^ cells.

We introduced the *dnaA46* allele into *seqA*_*S*36*A*_ and *seqA*_*S*36*D*_ cells by P1-transduction. The resultant strains are initiation proficient at 30°C (permissive temperature), but not at 42°C (non-permissive temperature) due to a reversible defect in nucleotide binding (Carr and Kaguni, 1996). Wild-type, *seqA*_*S*36*A*_ and *seqA*_*S*36*D*_ cells carrying the *dnaA46* allele were grown exponentially at 30°C. The average number of origins per cell for all three strains was close to 2 (Figures 3A–C) and the SeqA proteins were produced in similar amounts (Figure 3D). When cells were shifted to 42°C, initiations ceased whereas cells continued to grow and divide, resulting in most cells ending up having one fully replicated chromosome after 90 min (Figures 3A–C). Upon a shift back to 30°C, where the DnaA46 protein was reactivated, all cells initiated replication, i.e., doubled their origin content, within a short period of time. This round of initiation was followed by a period of ~20 min where all newly formed origins were inert to further initiation, after which replication initiation resumed (Figures 3A–C). This 20-min period represents the minimal time between successive initiations (von Freiesleben et al., 2000), and it did not differ between wild-type and *seqA* mutant cells (Figures 3A–C). SeqA deficient cells were previously shown to reinitiate frequently without this 20-min delay (von Freiesleben et al., 2000).

### Serine36 of SeqA is not phylogenetically conserved

We aligned SeqA amino acid sequences from the *Vibrio cholerae, Pasteurella multocida* PM70, *Haemophilus influenzae* Rd, *Yersinia enterocolitica, Serratia marcescens, Actinobacillus pleuropneumoniae* and *Glaesserella parasuis* with that of *E. coli* K12. All of these bacteria are known to carry *hipBA* genes. We looked for conservation of Ser36 along with the two flanking amino acids Phe35 and Ala37 (Table 4). None of these amino acids were conserved among the species with Ser36 showing the least degree of conservation. On the other hand Thr18, Ile21, and Ala25 which are instrumental in oligomerization of SeqA (Guarné et al., 2005), were completely conserved. For Arg116, Thr117, Arg118, Asn150, and Asn152 that make contact with the GATC sequence in DNA (Fujikawa et al., 2004) we also observed a high degree of conservation between species (Table 4). This may indicate a limited role of Ser36 for SeqA function.

**Table 4:** Serine 36 of *E. coli* SeqA is not conserved between bacterial species.

***Escherichia coli* K12**	**Thr18**	**Ile21**	**Ala25**	**Phe35**	**Ser36**	**Ala37**	**Arg116**	**Thr117**	**Arg118**	**Asn150**	**Asn152**
*Vibrio cholerae*	+	+	+	–	–	–	+	–	+	+	+
*Pasteurella multocida* PM70	+	+	+	+	+	–	+	+	+	+	+
*Haemophilus influenzae* Rd	+	+	+	–	–	–	+	+	+	+	+
*Yersinia enterocolitica*	+	+	+	+	–	+	+	+	+	+	+
*Serratia marcescens*	+	+	+	+	–	+	+	+	+	+	+
*Actinobacillus pleuropneumoniae*	+	+	+	–	–	–	–	–	+	+	+
*Glaesserella parasuis*	+	+	+	–	–	+	–	–	+	+	+

*The amino acid sequences of the E. coli K12, Vibrio cholera, Pasteurella multocida PM70, Haemophilus influenzae Rd, Yersinia enterocolitica, Serratia mercescens, Actinobacillus pleuropneumoniae and Glaesserella parasuis SeqA proteins were aligned. + and – indicate presence or absence of the indicated amino acid, respectively*.

## Discussion

Recently, it was shown that residue Ser36 in the SeqA protein is a target for phosphorylation by the serine-threonine kinase, HipA (Semanjski et al., 2018). HipA is mostly known for its role in bacterial persister formation through phosphorylation of a conserved serine, Ser239, residue in the GltX aminoacyl-tRNA synthetase, which inactivates the enzyme to arrest cell growth (Germain et al., 2013; Kaspy et al., 2013). Here, we wanted to determine whether Ser36 phosphorylation could alter SeqA activity. It was tempting to speculate that the Ser36 phosphorylation would activate SeqA, thereby enhancing its inhibition of replication initiation, which would contribute to shut down chromosomal replication in persister cells. SeqA was found to be endogenous phosphorylated in wild-type *E. coli* cells, and was revealed as a direct phosphorylation target of HipA *in vitro*. When the *hipA* gene was expressed from a p15A based plasmid, the fraction of wild-type SeqA found to be phosphorylated at residue Ser36 was ~7% following 95 min induction (Semanjski et al., 2018). It could be argued that this is a relative small fraction of the total SeqA protein. However, one should be aware that the actual phosphorylation status of SeqA may depend on the specific conditions provided. In the Semanjski study HipA expression was countered by the antitoxin HipB produced from the chromosome. The fraction of phosphorylated SeqA may therefore not reflect the fraction of SeqA being phosphorylated during an actual stress-induced situation where HipA becomes fully induced without HipB-mediated neutralization, and where the overall protein synthesis is affected. Also, it remains unknown whether all SeqA molecules present in the cell are actually available to HipA-mediated phosphorylation. The oligomerization domain of SeqA (residue 1–33) is located close to the HipA phosphorylation domain at residue Ser36 (see below), and hence it is not clear whether SeqA oligomers are available to phosphorylation, or whether only SeqA monomers become phosphorylated.

The Ser36 residue is located in the flexible linker between the N-terminal oligomerization domain and the C-terminal DNA binding domain (Chung et al., 2009). Neither of the phospho-impaired (S36A) nor the phospho-mimetic (S36D) SeqA proteins have any change in linker length nor are they affected in prolin or other amino acid residues suggested as most preferred in linker regions (George and Heringa, 2002), suggesting that changes in flexibility and hydrophobicity are non-significant upon phosphorylation of Ser36. This agrees well with the tertiary structural predictions of the SeqA, SeqA_S36A_, and SeqA_S36D_ proteins that indicated the mutations to cause minor structural changes to the linker region only, leaving the N- and C-terminal domains unaffected. This might explain our observations that function and activity of the SeqA mutant proteins seemed unaffected by the Ser36 mutations with respect to replication initiation control.

Although we have assumed that substituting a serine residue with a negatively charged amino acid, such as aspartic acid, imparts the negative charge associated with serine phosphorylation, caution should be taken as this is not always the case. The phospho-mimetic proteins may fail to recapitulate the true steric and charge-based nature of phosphorylation (Paleologou et al., 2008). Also, the “phosphorylation status” mimicked by phospho-mimetics is non-reversible, and hence cannot reflect the true state of phosphorylation-mediated protein modification. Therefore, the SeqA_S36D_ protein may deviate in activity from the phosphorylated wild-type SeqA protein.

However, because removal of the HipA kinase in wild-type cells revealed no replication phenotype, we find it unlikely that HipA-mediated Ser36 phosphorylation affects the activity of SeqA, at least with respect to its function in replication initiation, and at least under the conditions provided in this study. SeqA phosphorylation may therefore be an example of a silent phosphorylation. This has previously been observed for pepsin and ovalbumin, where serine phosphorylation did not affect protein activity, and the function of the phosphate group remained unknown (Johnson and Barford, 1993). The proposal that SeqA phosphorylation is silent is reinforced by the low degree of Ser36 conservation between *hipBA* carrying bacterial species compared to highly conserved amino acids crucial for oligomerization and DNA binding activity.

## Author contributions

LR, EG, and AL-O planned the experiments. LR, BK, and LK performed the experiments. LR, BK, LK, and AL-O analyzed data. LR, BK, and AL-O wrote the manuscript.

### Conflict of interest statement

The authors declare that the research was conducted in the absence of any commercial or financial relationships that could be construed as a potential conflict of interest.
